# PDE10A as a novel diagnostic and therapeutic target in cancer: insights and challenges

**DOI:** 10.3389/fonc.2025.1669157

**Published:** 2025-10-16

**Authors:** Sahar Ghoflchi, Ali Nakhaei, Farzaneh Abbasinezhad-Moud, Mohammad Jalili-Nik, Patrick J. Cimino

**Affiliations:** ^1^ Student Research Committee, Mashhad University of Medical Sciences, Mashhad, Iran; ^2^ Department of Clinical Biochemistry, Faculty of Medicine, Mashhad University of Medical Sciences, Mashhad, Iran; ^3^ Student Research Committee, MMS.C., Islamic Azad University, Mashhad, Iran; ^4^ Neuropathology Unit, National Institute of Neurological Disorders and Stroke, National Institutes of Health, Bethesda, MD, United States

**Keywords:** phosphodiesterase 10A, PDE10A, cyclic adenosine monophosphate, cyclic guanosine monophosphate, cancer

## Abstract

Phosphodiesterase 10A (PDE10A) is a dual-substrate enzyme that hydrolyzes both cyclic adenosine monophosphate (cAMP) and cyclic guanosine monophosphate (cGMP), playing a critical role in regulating intracellular signaling pathways. While its function has been extensively studied in the central nervous system, emerging evidence highlights its broader physiological and pathological relevance, including its involvement in cancer. Functionally, it modulates key signaling pathways such as cAMP/protein kinase A (PKA) and cGMP/protein kinase G (PKG), influencing cell proliferation, differentiation, and apoptosis. In cancer, PDE10A exhibits a context-dependent role. It functions as an oncogene in cancers such as colorectal, ovarian, gastric, and non-small cell lung cancers through overexpression and downstream activation of the Wnt/β-catenin, MAPK/ERK, and PI3K/AKT pathways. Pharmacological inhibition of PDE10A using selective inhibitors has demonstrated potent anti-tumor effects in preclinical models by restoring cyclic nucleotide levels and suppressing oncogenic signaling. Conversely, in glioblastoma (GBM), PDE10A acts as a tumor suppressor, and its knockdown promotes tumor progression via activation of the PI3K/AKT pathway. These findings showed the ability of PDE10A to be considered as a promising biomarker and therapeutic target in oncology; however, it is suggested to examine the tissue-specific expression of PDE10A, baseline cyclic nucleotide levels, cross-talk with other pathways, differences in the degree and duration of PDE10A suppression, and the interplay between PDE10A-mediated cyclic nucleotide signaling and compensatory oncogenic pathways for an effective therapy as observed in other PDEs family reviewed in this manuscript.

## 1 Introduction

Phosphodiesterase (PDE) 10A belongs to a family of at least eleven phosphodiesterase isoenzymes responsible for the hydrolytic degradation of the second messengers, cyclic adenosine monophosphate (cAMP) and/or cyclic guanosine monophosphate (cGMP). PDE10A, the sole gene product of this family, exhibits dual substrate specificity. Although it demonstrates a higher binding affinity for cAMP, it more efficiently hydrolyzes cGMP. Three protein-coding isoforms of PDE10A have been identified, including PDE10A1 and PDE10A19 that localize to the cytosol, and membrane-associated PDE10A2 (1–3).

The pharmacological inhibition of PDE10A has garnered increasing interest in both academic and industrial research due to its potential to elevate intracellular cAMP and/or cGMP levels. Such modulation holds therapeutic promise across a range of disorders involving both the central and peripheral nervous systems, as well as in various malignancies (4). By modulating intracellular levels of cyclic nucleotides, such as cAMP and cGMP, PDE10A can influence key processes, including cell proliferation, apoptosis, and metastasis. Recent studies suggest that PDE10A is overexpressed in specific cancer types and may contribute to tumor progression, making it a potential biomarker and therapeutic target (5–12).

Despite the growing evidence linking PDE10A to cancer biology, most existing reviews focus primarily on its role in neurological disorders, and only limited literature addresses its function in oncogenesis (13, 14). Given the recent studies highlighting PDEs involvement in tumor growth, this review aimed to explore the structure, signaling, and significant roles of the Phosphodiesterase family in cancer with a focus on the dual role activity of PDE10A in various cancers and its potential to be used as a biomarker for diagnosis, prognosis, or as a therapeutic target.

## 2 Phosphodiesterase family: structure, signaling, and roles in cancer

The PDEs constitute a superfamily of enzymes that regulate intracellular levels of cAMP and cGMP by catalyzing their hydrolysis to 5′ AMP and 5′ GMP, respectively (15, 16). Mammalian PDEs are classified into 11 subfamilies encoded by 21 distinct genes, many of which produce multiple splice variants with unique subcellular localizations and regulatory roles (15, 17). These enzymes are categorized based on substrate specificity, including cAMP-specific (PDE4, PDE7, PDE8), cGMP-specific (PDE5, PDE6, PDE9), and dual-specificity PDEs that hydrolyze both cyclic nucleotides (PDE1, PDE2, PDE3, PDE10, and PDE11), though individual members often display a higher affinity for one nucleotide (15, 17). Substrate recognition is modulated by a conserved glutamine switch, which regulates purine ring binding within the catalytic domain (18). While their catalytic and regulatory domains are conserved, isoform-specific differences, particularly in the N- and C-terminal regions, define the subcellular targeting and functional specificity of each PDE (17, 19). The expression of PDEs is variable and widely distributed across tissues, including the brain, where each isoform shows distinct spatiotemporal expression patterns, emphasizing their role in fine-tuning cyclic nucleotide signaling (20, 21). Although the primary aim of this review is to explore PDE10A as a diagnostic and therapeutic target in cancer, we first provide a brief overview of the structural characteristics and oncological relevance of other PDE isoforms to show and compare the dual role and expression of PDE10A in cancers.

### 2.1 Phosphodiesterase 1

Phosphodiesterase 1 (PDE1) is a calmodulin- and Ca²-dependent enzyme family comprising three subtypes, including PDE1A, PDE1B, and PDE1C. PDE1A, PDE1B, and PDE1C are encoded by genes located on chromosomes 2q32.1, 12q13, and 7p14.3, respectively. Alternative splicing generates multiple isoforms, including ten for PDE1A, two for PDE1B, and five for PDE1C. PDE1 isoforms are broadly expressed in various tissues, including the heart, brain, lung, and smooth muscle, with distinct distribution patterns and physiological roles (17). Uniquely, PDE1 enzymes are regulated by two Ca²^+^/calmodulin binding domains and two phosphorylation sites at the N-terminus (22). These enzymes hydrolyze both cAMP and cGMP with isoform-specific nucleotide affinities. PDE1A and PDE1B exhibit higher affinity for cGMP. However, PDE1C hydrolyzes cAMP and cGMP with comparable efficiency (23).

Functionally, PDE1 has been implicated in the regulation of cell proliferation in various malignancies. It is a specific pharmacological target of differentiation-inducing factor 1, which inhibits cancer cell proliferation by competitively blocking cAMP binding to PDE1 in a dose-dependent manner (24). In melanoma cells, PDE1C is overexpressed, and its inhibition by vinpocetine significantly reduces tumor growth (25). Natural compounds such as curcumin and thymoquinone have been shown to target PDE1, leading to apoptosis and growth inhibition in melanoma and leukemia cells, respectively, through modulation of epigenetic and cell cycle-related markers (26, 27). Specifically, thymoquinone suppresses PDE1A expression and inhibits UHRF1 via a p73-dependent mechanism, suggesting potential application in acute lymphoblastic leukemia (ALL) therapy (**Table 1**) (27). Another study by Zhang et al. showed the importance of PDE1A as a significant promoter of Non-small cell lung cancer (NSCLC) metastasis through regulating exosome release and activating the STAT3 pathway by interacting with YTHDF2, suggesting PDE1A as a potential therapeutic target for metastatic disease (28).

**Table 1:** Different PDEs targeted or used as biomarkers in each cancer.

Family	Substrate	Regulation	Cancer	Main function	Reference
PDE1	cAMP/cGMP	Ca^2+^/calmodulin	-Melanoma -GBM -Leukemia -NSCLC -Osteosarcoma	-PDE1A & PDE1C act as oncogenes by affecting key pathways like UHRF1, p73, cAMP, and STAT3 -PDE1B acts as a tumor suppressor	(25, 27–30)
PDE2	cAMP/cGMP	cGMP stimulated	-Melanoma -colon carcinoma - osteosarcoma - HCC	-PDE2 Promotes tumor progression by reducing cAMP levels, affects TFAM stability -Acts as a tumor suppressor in HCC by altering mitochondrial morphology and ATP content	(31–35)
PDE3	cAMP/cGMP	Phosphorylation/cGMP inhibited	- PDAC - breast cancer	-Inhibition of PDE3 has anticancer effects by stabilizing the PDE3A-SLFN12 complex, suppress cancer stem cells, halt tumor growth and metastasis, and modulate cAMP/PKA and NFκB signaling pathways	(36–39)
PDE4	cAMP	Phosphorylation/cAMP-specific UCR1/UCR2 regions	-Glioma -lung cancer -prostate cancer –CRC -CLL -HCC - gastric cancer -UBC	-PDE4 isoforms exhibit diverse, context-dependent roles in cancer -Acting as tumor suppressors (e.g., suppressing migration in glioma via cAMP-p53) -Act as an oncogene (e.g., driving proliferation in lung cancer via cAMP-PKA/EPAC-HIF, and in other cancers through pathways like PI3K/AKT/MYC, β-catenin, and FAK/RACK1/PDE4D5/Rap1), - PDE4 inhibition can induce apoptosis in CLL and colorectal cancer	(40–48)
PDE5	cGMP	Phosphorylation/cGMP-specific	-PTC -Melanoma -Prostate cancer -PTCs - Lung cancer - Breast cancer - CRC -HCC - Esophageal adenocarcinoma -Gastrointestinal/genitourinary cancers	-PDE5 is overexpressed in numerous cancers -Its inhibition exerts anti-tumor effects through modulating cGMP/PKG signaling; disrupts cancer stem cell maintenance; impairs DNA repair pathways to sensitize cells to chemotherapy; enhances drug uptake via endocytosis; re-programs the tumor microenvironment by blocking pro-tumor inflammatory signals from fibroblasts and MDSCs; and promotes autophagy while inhibiting oncogenic pathways like IL-6/JAK/STAT3.	(49–63)
PDE6	cGMP	Phosphorylation/cGMP-specific	-Melanoma -Breast cancer -CRC -HCC	-PDE6 plays an oncogenic role in several cancers by disrupting cyclic nucleotide signaling, Wnt5a-Frizzled-2 pathway, and ERK activation -PDE6H knockout suppresses mTORC1 signaling and mitochondrial function, and induces cell cycle arrest.	(64–67)
PDE7	cAMP	Rolipram-insensitive	-CLL -MCL -EC -CRC -GBM - Breast cancer -HCC - ccRCC cells	- PDE7 isoforms exhibit complex, cancer-type-specific mechanisms and roles as either oncogenic promoters or tumor suppressors - Oncogenic mechanisms involve triggering mitochondrial depolarization, suppressing tumor-suppressive microRNAs (e.g., miR-1/133a), regulating the EMT process, and activating the PI3K/AKT signaling pathway. -Anti-tumorigenic capacities, as its knockdown increases cell viability and migration	(68–76)
PDE8	cAMP	cAMP-specific	Kidney cancer	-Promotes cancer progression by enhancing ERK pathway activation through direct interaction with Raf-1 kinase.	(77)
PDE9	cGMP	cGMP-specific	Breast cancer	- Enhances cancer cell growth. - Pharmacological inhibition reduces proliferation and induces apoptosis	(78, 79)
PDE10	cAMP/cGMP	cAMP-inhibited	Colon cancer, NSCLC	-Enhances tumor cell proliferation, while inhibition activates cGMP/PKG signaling and blocks β-catenin nuclear translocation. -In NSCLC, PDE10A inhibition reduces proliferation by downregulating both Ras and Wnt pathways	(6, 80)
PDE11	cAMP/cGMP	cGMP-activated	AIMAH, Carney complex	-Mutations may enhance cAMP signaling, contributing to tumorigenesis	(81, 82)

Phosphodiesterase: PDE, Acute lymphoblastic leukemia: ALL, Glioblastoma: GBM, Schlafen 12: SLFN12, Chronic lymphocytic leukemia: CLL, Papillary thyroid carcinomas: PTCs, Myeloid-derived suppressor cells: MDSCs, Mantle cell lymphoma: MCL, Endometrial cancer: EC, Non-small cell lung cancer: NSCLC, Macronodular adrenocortical hyperplasia: AIMAH, UBC: urinary bladder cancer, CRPC: Castration-resistant prostate cancer, PTCs: Papillary thyroid carcinomas, NSCLC: Non-small cell lung cancer, CRC: Colorectal carcinoma, HCC: Hepatocellular carcinoma, PDAC: Pancreatic ductal adenocarcinoma, MDSCs: Myeloid-derived suppressor cells

In IDH-wildtype GBM, elevated PDE1C expression correlates with poor prognosis (83). Genetic silencing of PDE1C using siRNAs significantly disrupts GBM cell proliferation, migration, and invasion, highlighting its potential as a therapeutic target (**Table 1**) (29). Collectively, these findings highlight the therapeutic promise of targeting PDE1 in cancer treatment. However, the development of highly selective and potent PDE1 inhibitors remains a significant unresolved challenge (84).

In contrast to most PDE1 subtypes, a study by Chen et al. investigates the role of PDE1B in osteosarcoma, suggesting that PDE1B is a tumor suppressor gene that prevents osteosarcoma from escaping the immune system (30). This study highlighted the importance of considering dual role activity of PDE1 in cancers, requiring further study.

### 2.2 Phosphodiesterase 2

The Phosphodiesterase 2 (PDE2) is a dual-substrate enzyme that hydrolyzes both cAMP and cGMP, with half-maximal velocities (Km) of approximately 30 µM and 10 µM, respectively (85, 86). It is uniquely stimulated by cGMP, which enhances cAMP hydrolysis by up to six-fold, earning it the designation cGMP-stimulated PDE (85, 86). PDE2 is encoded by a single gene, *PDE2A*, which gives rise to three isoforms, including PDE2A1, PDE2A2, and PDE2A3 (87). It is broadly expressed in human tissues such as the adrenal gland, heart, lung, liver, platelets, and endothelial cells (88). Several selective inhibitors have been developed, including EHNA, BAY 60-7550, oxindole derivatives, IC933, PDP, and ND7001; however, these have not yet been evaluated in clinical cancer therapy (89–91).

In the context of cancer biology, PDE2 has been implicated in tumor progression. Notably, specific tumor cells, such as malignant melanoma and colon carcinoma, exhibit reduced cAMP levels, where cAMP serves as a negative regulator of proliferation (31). Zhao et al. showed that mitochondrial calcium (Ca^2+^) could activate PDE2, acting as an inhibitor of PKA, which affects mitochondrial transcription factor A (TFAM) stability and increases CRC growth (32).

In malignant melanoma cells, inhibition of PDE2 using EHNA or PDE2A-targeted siRNAs suppressed both cell growth and invasion, suggesting a functional role for PDE2 in tumor aggressiveness (92). It has also been observed that topical application of erythro-9-(2-hydroxy-3-nonyl) adenine hydrochloride (EHNA hydrochloride) on a mouse model of ultraviolet light B (UVB)-induced skin carcinogenesis, a PDE2 inhibitor increases apoptosis and attenuates tumor formation (33). The exclusive expression of mutant PDE2A2 isoforms in these cells indicates that PDE2A2 may serve as a novel therapeutic target in malignant melanoma (**Table 1**) (93). In addition to skin cancer, Murata et al. reported that EHNA and 8-bromo-cAMP PDE2 inhibitors could decrease proliferation and migration of HOSM-1 osteosarcoma cells (34).

In contrast to these findings, Chen et al. report that PDE2A has an inverse correlation with AFP level, immune function, vascular invasion, grade, and stage of HCC patients. In addition, overexpression of PDE2A in two HCC cell lines (HLF and SNU-368) caused inhibition of invasion, colony formation, migration, and proliferation, possibly via a change in mitochondrial morphology and ATP content (35). This inconsistency has been reported to be likely related to the variation in predominant PDE isoforms across different tissues, as well as unidentified mechanisms through which of these isoforms regulate cellular behavior (35).

### 2.3 Phosphodiesterase 3

Phosphodiesterase 3 (PDE3) is a dual-specificity enzyme that hydrolyzes both cAMP and cGMP, with a higher affinity for cGMP. Consequently, cGMP acts as a potent competitive inhibitor of cAMP hydrolysis (94). The PDE3 family consists of two genes, *PDE3A* and *PDE3B*. *PDE3A* is expressed in three splice variants, including PDE3A1 (136 kDa), PDE3A2 (118 kDa), and PDE3A3 (94 kDa), whereas no splice variants have been identified for *PDE3B* (95). Structurally, PDE3 enzymes contain N-terminal hydrophobic membrane-association regions and a unique 44-amino acid insertion within the catalytic domain (96). Multiple phosphorylation sites in the N-terminal region confer diverse regulatory functions (97).

PDE3 is widely expressed in cardiac and vascular myocytes, brain, liver, adipose tissue, pancreatic β-cells, endothelium, epithelium, oocytes, and platelets (98). Functionally, PDE3A regulates cardiac contractility, platelet aggregation, smooth muscle contraction, oocyte maturation, and renin secretion, whereas PDE3B is more involved in insulin signaling and cell proliferation (99, 100). Several potent and selective PDE3 inhibitors, including cilostamide, cilostazol, and olprinone, have been developed (101).

Recent studies have highlighted PDE3A as both a biomarker and a potential therapeutic target in cancer. A novel mechanism has been described for the small molecule DNMDP (6-(4-(diethylamino)-3-nitrophenyl)-5-methyl-4,5-dihydropyridazin-3(2H)-one), which acts through a gain-of-function allosteric mechanism. DNMDP stabilizes the interaction between PDE3A and Schlafen 12 (SLFN12), inducing cytotoxicity in cancer cells with elevated expression of both proteins (**Table 1**) (36, 37). Another study on pancreatic ductal adenocarcinoma (PDAC) showed that combining the epigallocatechin-3-O-gallate with a PDE3 inhibitor dramatically suppressed cancer stem cells (CSCs) and halted tumor growth and metastasis *in vivo* (38). In addition, PDE3A was shown to be able to suppress cAMP/PKA and induce NFκB signaling pathway, causing expression of the stem cell marker OCT4 and cancer stemness in breast cancer. Inhibition of PDE3A with cilostazol could reduce metastasis and suppress tumor growth in xenograft breast cancer models (39).

### 2.4 Phosphodiesterase 4

The Phosphodiesterase 4 (PDE4) represents the largest and one of the earliest identified PDE families, characterized by its specificity for cAMP hydrolysis, with a Km ranging between 2–4 µM (102). It comprises four genes including *PDE4A*, *PDE4B*, *PDE4C*, and *PDE4D* that located on chromosomes 19p13.2, 1p31, 19p13.11, and 5q12, respectively (103). Alternative splicing of the N-terminal region results in the generation of multiple isoforms in long, short, and super-short forms, based on the presence or absence of upstream conserved regions (UCR1 and UCR2). These UCRs regulate isoform activity through phosphorylation by PKA and ERK, and influence PDE4 dimerization (104).

PDE4 is broadly expressed in various tissues, including the brain, liver, heart, lungs, smooth muscle, endothelial cells, and immune cells. Functionally, it modulates critical physiological and pathological processes, including neural function, immune cell activation, vascular remodeling, fertility, and cardiac activity (104). Notably, PDE4 demonstrates the highest cAMP-hydrolyzing activity in 41 out of 60 examined tumor cell lines (105). It has been implicated in the pathogenesis of numerous brain tumors, including glioblastoma, medulloblastoma, and ependymoma, and may serve as a therapeutic target in these malignancies (106).

In glioma, *PDE4C* suppressed migration and induced apoptosis through the cAMP-p53 axis, and its expression could be epigenetically silenced by promoter hypermethylation, suggesting a role as a glioma biomarker (40). Additionally, PDE4 is upregulated in lung cancer and contributes to tumor progression via the cAMP–PKA/EPAC–HIF pathway, while its inhibition attenuates tumor cell proliferation (41). TGF-β1-mediated PDE4 upregulation promotes epithelial–mesenchymal transition (EMT) in lung epithelial cells, identifying PDE4 as a target for mitigating EMT-related lung pathologies (**Table 1**) (107). EMT was also enhanced in hepatocellular carcinoma following ectopic expression of PDE4a (42).

In prostate cancer, PKA-mediated phosphorylation of PDE4 may enhance tumor growth (43). In addition, the PDE4B/PKA pathway plays a key role in the progression of androgen-dependent prostate cancer to castration-resistant prostate cancer (CRPC), which is non-responsive to docetaxel and has a poor prognosis (44). Furthermore, PDE4B is upregulated by oncogenic KRAS in CRC, and its inhibition by rolipram or shRNA induces apoptosis and restores epithelial polarity, underscoring its value as both a therapeutic and prognostic marker (45). PDE4B is reported to be able to promote immune infiltration of the tumor microenvironment (TME) and increase the clonal formation, proliferation, migration, and invasion of gastric cancer via the PI3K/AKT/MYC pathway (46). PDE4B is also able to promote proliferation, migration, invasion, and EMT of urinary bladder cancer (UBC) via the β-catenin pathway, while its inhibition by rolipram reverses these effects (47).

Moreover, the higher expression of PDE4, along with exchange protein 1 directly activated by cAMP (Epac1), has been reported in rectal carcinoma (108). The FAK/RACK1/PDE4D5/Rap1 axis facilitates tumor cell adhesion and migration through localized cAMP degradation (109). In chronic lymphocytic leukemia (CLL), PDE4 inhibition promotes apoptosis via activation of PP2A and dephosphorylation of Bcl-2 family members (48). Another study on advanced B-cell malignancies reported the efficacy of roflumilast as a PDE4 inhibitor in combination with prednisone in the treatment of patients with B-cell malignancies (110).

Another study by Mukherjee et al. reported a higher level of PDE4 in breast CSCs compared to normal stem cells. Inhibition of PDE4 by rolipram in breast CSCs caused cell cycle arrest and apoptosis via antagonizing the PI3K/AKT/mTOR pathway and noncanonical activation of mTOR. Moreover, this study showed that the combination of Rolipram with paclitaxel caused a synergistic effect and eradicated breast CSCs (111). An increased expression of PDE4D in MCF-7 and T47D resistant cells to tamoxifen, a crucial hormonal therapy for ER-positive breast cancer, has been reported to be related to worse survival in tamoxifen-treated patients with breast cancer. Inhibition of PDE4D by dipyridamole and Gebr-7b or siRNAs could have restored tamoxifen sensitivity via activating cAMP/ER stress/p38-JNK signaling and induction of apoptosis (112). Upregulation of PDE4D was also seen in pancreatic ductal adenocarcinoma (PDAC) tumors with higher clinical progression and poor prognosis (113). Another study by Cao et al. also reported the involvement of PDE4D in clear cell renal cell carcinoma (ccRCC). They showed that targeting PDE4D with roflumilast or its knockout using CRISPR/Cas9 reduces the progression of ccRCC cells and enhances the apoptotic effect of sorafenib via attenuating MAPK/ERK signaling in a CRAF-dependent manner (114). Overall, PDE4 has been implicated in a wide range of cancers, including melanoma, DLBCL, liver, and colon cancers, positioning it as a promising target for novel anti-tumor strategies (**Table 1**) (84).

### 2.5 Phosphodiesterase 5

The Phosphodiesterase 5 (PDE5) is a cGMP-specific hydrolase encoded by a single gene, *PDE5A*, located on chromosome 4q27. In humans, it produces three N-terminal splice variants, including PDE5A1, PDE5A2, and PDE5A3 (115). The N-terminal domain harbors two GAF domains, comprising GAF-A, which mediates allosteric binding of cGMP, and GAF-B, which modulates the affinity of GAF-A for cGMP (116). PDE5 is predominantly expressed in platelets, vascular smooth muscle, brain, lung, heart, kidney, and skeletal muscle, with PDE5A1 and PDE5A2 widely distributed across tissues, while PDE5A3 is confined to vascular smooth muscle (117). Functionally, PDE5 is a critical regulator of vascular tone, particularly in penile and pulmonary tissues, modulates NO-cGMP signaling in platelets, and may influence cGMP signaling in the brain (118).

Dysregulated PDE5 expression has been reported in several malignancies, including bladder, prostate, breast, brain, colorectal, oral, and NSCLC, as well as in chronic lymphocytic leukemia (49, 118, 119). Intriguingly, oncogenic BRAF V600E downregulates *PDE5A* in melanoma, leading to elevated cGMP levels and increased invasiveness (50). Moreover, the PDE/cGMP/PKG axis is essential for maintaining the stemness of PC3-derived cancer stem cells, and combining PDE5 inhibitors with chemotherapeutics effectively impedes prostate cancer progression and metastasis (**Table 1**) (51).

The scientists investigated the association between PDE5 inhibitor and prostate cancer risk in the Reduction by Dutasteride of Prostate Cancer Events (REDUCE) trial. They found PDE 5 inhibitor use was not associated with decreased prostate cancer diagnoses on *post-hoc* analysis of REDUCE. Notably, they revealed that in North American men, who had much higher baseline use of PDE5 inhibitors, this treatment was associated with an inverse trend of prostate cancer diagnosis, but did not reach statistical significance (120). Another study on prostate cancer revealed that PDE5 inhibitors sildenafil and vardenafil, but not tadalafil, sensitize CRPC cells to doxorubicin and other topoisomerase II inhibitors. This combination treatment enhances apoptosis in a PDE5-independent mechanism by impairing two key DNA repair pathways, including homologous recombination and non-homologous end joining (52).

PDE5 is also reported in human papillary thyroid carcinomas (PTCs), especially in those with a BRAF mutation. The PDE5 inhibitors sildenafil and tadalafil were found to reduce the proliferation and migration of thyroid cancer cells *in vitro*, suggesting that targeting PDE5 may be a potential treatment strategy for PTCs (49). PDE5 inhibitors dipyridamole, vardenafil, and/or sildenafil could also increase the uptake of anti-cancer drugs in lung cancer cells by enhancing endocytosis both *in vitro* and *in vivo*. This effect significantly improved the anti-tumor efficacy of trastuzumab in a lung cancer xenograft nude mice model (53).

It has also been reported that mRNA and protein expression of PDE5 increased in human breast cancer patients, enhancing the tumor-stimulatory activities of fibroblasts and decreasing the survival of patients. Expression of PDE5 in mouse embryonic fibroblasts (MEFs) also increases cell proliferation, motility, and invasion, making it a potential target for cancer (54). Another study used Tadalafil as a potent PDE-5 inhibitor against N-methyl-N-nitrosourea-induced mammary gland carcinogenesis and found that a PDE-5 inhibitor could restore all biological markers to normal through blockade of DuCLOX signaling and attenuation of mitochondrial-oxidative stress (55). Catalano et al. also reported that elevated PDE5 expression is linked to more aggressive breast cancer subtypes and shorter patient survival, and PDE5 overexpression enhances cancer cell invasion and motility by activating Rho GTPase signaling (56).

PDE5 inhibitors were reported to be able to decrease the risk of CRC in patients diagnosed with benign colorectal neoplasm (57). PDE5 inhibitors also could prohibit the development and progression of aflatoxin-induced Hepatocellular carcinoma in rats (58). PDE5 is also expressed in myeloid-derived suppressor cells (MDSCs), where it contributes to immune evasion by promoting cytokine secretion, ROS production, and upregulation of nitric oxide synthase and arginase. Inhibiting PDE5 with sildenafil reduced MDSC infiltration and suppressed inflammation-driven colon tumorigenesis in preclinical models (59, 60). Additionally, cGMP signaling via PKG2 promotes expression of antioxidant genes through activation of FOXO transcription factors, particularly in the colonic epithelium. It highlights the therapeutic potential of PDE5 inhibitors in targeting redox and immune pathways (**Table 1**) (121). Moreover, P. Sharpe et al. reported that PDE5 inhibitors could inhibit the tumor-promoting function of cancer-associated fibroblasts and increase the efficacy of chemotherapy in esophageal adenocarcinoma (61). Using PDE5 inhibitors is also reported to be useful in improving the effect of chemotherapy agents against gastrointestinal/genitourinary cancers by promoting autophagy and inducing DNA damage (62). Another type of study found that the PDE5 inhibitor sildenafil suppresses gastric tumor growth by activating PKG, which leads to the degradation of c-MYC and the suppression of the IL-6/JAK/STAT3 signaling pathway (63). Collectively, these findings position PDE5 as a promising prognostic marker and a novel therapeutic target in various cancers.

### 2.6 Phosphodiesterase 6

The Phosphodiesterase 6 (PDE6), primarily known as the photoreceptor PDE, consists of several subunits encoded by distinct genes comprising *PDE6A* (5q31.2–34), *PDE6B* (4p16.3), and *PDE6C* (10q24), along with regulatory or accessory subunits *PDE6D* (2q35-q36), *PDE6G* (17q25), and *PDE6H* (12p13) (122). PDE6 is abundantly expressed in the photoreceptor outer segments of the mammalian retina and the pineal gland (123). In rod cells, the active enzyme is a heterodimer of PDE6A (α subunit) and PDE6B (β subunit), while in cone cells, the enzyme forms a homodimer of PDE6C (α′ subunit) (122). Each subunit contains two N-terminal GAF domains, including GAF-A and GAF-B, and a C-terminal catalytic domain. The GAF-A domain serves as a high-affinity cGMP-binding site and is likely critical for dimerization (124). While PDE6 exhibits high specificity for cGMP at low concentrations, it can also hydrolyze cAMP at elevated levels (123).

PDE6 was the first PDE family associated with genetic diseases, with mutations in the α and β subunits implicated in stationary night blindness and various forms of retinitis pigmentosa (125). Beyond the retina, PDE6 has been involved in cancer biology. In melanoma cells, deregulated activation of PDE6 occurs through a Wnt5a-Frizzled-2 signaling cascade, which reduces cGMP levels and increases intracellular calcium mobilization, thereby influencing cellular homeostasis (64). Furthermore, PDE6 may have oncogenic relevance in breast cancer. Epidemiological data indicate a link between artificial light at night and increased breast cancer risk (**Table 1**) (65). Microarray analyses of breast cancer cell lines and patient tissues revealed significant expression of *PDE6B*, *PDE6C*, and *PDE6D*, with minimal to no expression of *PDE6A*, *PDE6G*, and *PDE6H*. Immunohistochemistry confirmed PDE6B protein presence in multiple patient samples and MCF-7 breast cancer cells (126). Moreover, PDE6H, the inhibitory (or gamma) subunit of the cone-specific cGMP phosphodiesterase, has been identified as a key controller of cancer cell growth. Knockout of PDE6H increased cGMP levels, reduced mTORC1 signaling, induced cell cycle arrest, suppressed mitochondrial function, and slowed tumor growth in a xenograft model. This study also reported that treatment with the PDE5/6 inhibitor sildenafil decreases CRC tumor growth and improves survival (66). Moreover, PDE6D, as the delta subunit of rod-specific photoreceptor cGMP phosphodiesterase, is significantly overexpressed in hepatocellular carcinoma and correlates with advanced tumor stages and ERK activation. PDE6D expression was also induced by TGF-β1 and was overexpressed in sorafenib-resistant cells. Functionally, PDE6D depletion reduced cancer cell proliferation and migration, and conferred resistance to the chemotherapy drug sorafenib (67). These findings establish PDE6D as a key contributor to tumor progression and chemoresistance, marking it as a promising new therapeutic target for cancer.

### 2.7 Phosphodiesterase 7

The Phosphodiesterase 7 (PDE7) is a cAMP-specific enzyme with high affinity for its substrate, particularly at low concentrations (127). It comprises two genes, including *PDE7A*, located on chromosome 8q13, and PDE7B, on chromosome 6q23–24. PDE7A encodes three splice variants, including PDE7A1, PDE7A2, and PDE7A3, while *PDE7B* encodes four variants. Notably, the N-terminal region of PDE7 lacks a known regulatory domain, although it contains consensus sites for PKA phosphorylation. PDE7 is widely expressed at both mRNA and protein levels in various immune cells, implicating a possible role in T-lymphocyte activation (128).

In the nervous system, PDE7B1 is activated via the cAMP/PKA/cAMP response element-binding protein (CREB) signaling axis in striatal neurons and may be involved in memory regulation. It is suggested to have potential as a therapeutic target in neurodegenerative conditions such as Parkinson’s and Huntington’s diseases (129). In oncology, PDE7 expression levels serve as a prognostic indicator in hematologic malignancies. For example, *PDE7B* is upregulated 23-fold in CLL and 21-fold in mantle cell lymphoma compared to normal peripheral blood mononuclear cells, indicating its value as a molecular target for therapy (68, 69). Additionally, overexpression of PDE7A in endometrial cancer (EC) cells facilitates cancer cell migration and invasion through the suppression of the miR-1/133a microRNA cluster (**Table 1**) (70). Lowering PDE7A expression with miR-23b or silencing PDE7A could also reduce the migration and invasion abilities of colon cancer cells (SW620 and SW480 cells) (71). Pharmacological inhibition of PDE7 using BRL-50481 enhances cAMP-PKA-dependent apoptosis in CLL cells, further supporting its therapeutic relevance (69). Elevated PDE7B expression has been reported in GBM cases, negatively impacts patient survival, and promotes the expansion of stem-like cancer cells and increased tumor aggressiveness *in vitro* and *in vivo* (72). Zhang et al. also discovered that suppressing PDE7B using miR-200c could inhibit the proliferation and progression of triple-negative breast cancer cells (73). In addition, inhibiting PDE7 using BRL-50481, IR-202, and IR-284, increasing intracellular cAMP levels, which triggers mitochondrial depolarization and the release of cytochrome c, caused apoptosis of chronic lymphocytic leukemia (CLL) (74). These findings demonstrate that PDE7B acts as an oncogene, increasing the progression of tumors, and pharmacological inhibition of PDE7B could be beneficial.

In contrast, Du et al. reported that PDE7B is significantly hypermethylated and downregulated in Hepatocellular carcinoma tissues. PDE7B expression has been reported to be correlated with poor prognosis and recurrence in HCC patients. Restoring PDE7B expression in hepatocellular carcinoma cell lines inhibited tumor proliferation and metastasis by regulating the EMT process and inhibition of the PI3K/AKT signaling pathway (75). Similarly, Sun et al. showed that PDE7B was downregulated in ccRCC cells, and knockdown of PDE7B could increase cell viability and migration, suggesting PDE7B has anti-tumorigenic capacity (76). This controversy may be related to the method used in these studies, which employed acute (pharmacological inhibition) or chronic (using stable overexpression or stable knockout) approaches, yielding different results. However, investigating the genetic and expression profiles of patients seems necessary before any intervention on PDE7 expression, bringing to us the importance of personalized medicine.

### 2.8 Phosphodiesterase 8

The Phosphodiesterase 8 (PDE8) is a cAMP-specific enzyme characterized by exceptionally high substrate affinity compared to other PDE isoforms. It comprises two homologous genes, *PDE8A* (chromosome 15q25.3) and *PDE8B* (chromosome 5q13.3), with *PDE8A* generating five splice variants (PDE8A1-PDE8A5) (130). PDE8A is broadly expressed, with predominant localization in the testis and T lymphocytes, whereas PDE8B is mainly found in the brain and thyroid (131). PDE8 plays essential roles in diverse physiological processes, including T-cell activation, Leydig cell steroidogenesis, spermatogenesis, thyroid hormone synthesis, and cardiac regulation (132). Although its N-terminal region contains REC and PAS domains, the precise regulatory mechanisms remain poorly understood (133).

Clinically, two PDE8B mutations (H391A and P660L) have been associated with ACTH-independent macronodular adrenocortical hyperplasia (AIMAH) and non-secreting adrenocortical carcinomas (ACCs). These associations suggest a potential role for PDE8B in tumorigenesis and increased susceptibility to ACCs (134). Furthermore, PDE8A interacts with Raf-1 kinase with high picomolar affinity via amino acids 454-465, thereby promoting Raf-1 phosphorylation and enhancing ERK pathway activation. Disruption of the PDE8A/Raf-1 interaction using a synthetic peptide suppressed ERK signaling and the cellular response to EGF. Overexpression of a dominant-negative, catalytically inactive PDE8A1 mutant resulted in elevated Raf-1 phosphorylation at the inhibitory S259 site (77). Additionally, genetic deletion of PDE8 in *Drosophila melanogaster* decreased basal ERK activity and increased susceptibility to stress-induced mortality. These findings highlight the PDE8A/Raf-1 signaling complex as a promising therapeutic target in cancer (**Table 1**) (77).

### 2.9 Phosphodiesterase 9

The Phosphodiesterase 9 (PDE9) is a cGMP-specific enzyme with the highest affinity for cGMP among all PDE isoforms. The human *PDE9A* gene, located on chromosome 21q22.3, gives rise to 21 known splice variants (135). Unlike other PDEs, PDE9A lacks GAF domains or other defined regulatory motifs in its N-terminal region. PDE9A is predominantly expressed in the spleen, intestine, and brain, with particularly high levels in Purkinje neurons and the cerebellum (136). Functionally, selective inhibition of PDE9A by BAY 73–6691 has been shown to enhance cognitive performance, improving learning and memory in rodent models (137, 138).

In oncological contexts, elevated PDE9A expression has been observed in malignant breast tumors compared to normal tissues (78). Treatment of MCF-7 and MDA-MB468 breast cancer cells with BAY 73–6691 led to a dose- and time-dependent reduction in cell proliferation and an increase in apoptosis, highlighting PDE9A as a promising therapeutic target in breast cancer (**Table 1**) (79).

### 2.10 Phosphodiesterase 10

The PDE10 is encoded by the *PDE10A* gene located on chromosome 6q26 and exists in multiple isoforms, with PDE10A1 and PDE10A2 being the most prominent variants in humans (139, 140). PDE10 isoforms contain two GAF domains and hydrolyze both cAMP and cGMP, although they exhibit a higher affinity for cAMP; notably, cAMP binds specifically to the GAF-B domain (141). PDE10A expression is predominantly localized in the brain, particularly in the striatum, as well as in the thyroid and pituitary glands, suggesting a role in modulating cGMP signaling involved in learning and memory processes (142).

PDE10A has also been implicated in several malignancies, including lung, breast, and colon cancers (80, 143, 144). Elevated expression of PDE10A has been detected in colon tumor cells compared to normal colonocytes, and siRNA-mediated knockdown of PDE10A significantly inhibited tumor cell proliferation (144, 145). Additionally, pharmacological inhibition of PDE10A suppressed colon tumor growth by activating cGMP/PKG signaling, which in turn blocked nuclear translocation of β-catenin (6). In NSCLC, PDE10A is overexpressed, and its inhibition via siRNA or selective inhibitors suppressed cell proliferation by simultaneously downregulating Ras and Wnt signaling pathways (**Table 1**) (80). These findings support PDE10A as a potential therapeutic target in certain cancer types.

### 2.11 Phosphodiesterase 11

The Phosphodiesterase 11 (PDE11) is a dual-substrate enzyme capable of hydrolyzing both cAMP and cGMP with comparable affinities (146). It is encoded by a single gene, *PDE11A*, located on chromosome 2q31.2, and gives rise to four splice variants (*PDE11A1–PDE11A4*) that differ in their N-terminal regions due to distinct transcriptional start sites (147). PDE11A expression has been detected in various human tissues, including skeletal muscle, prostate, testis, salivary and thyroid glands, and liver (148). Functional studies in knockout mice suggest a role for PDE11A in sperm development and function, as well as in the pathogenesis of testicular and adrenal hyperplasia, Cushing disease, and certain psychiatric conditions (149, 150).

PDE11A has also emerged as a potential target in oncology. It is notably expressed in several malignancies, including renal, prostate, colon, lung, and breast cancers (151). In prostate cancer, inactivating mutations in *PDE11A* correlate with reduced protein expression, although the contribution of these mutations to cancer susceptibility remains unclear (152). The PDE11A4 isoform has been implicated as a predisposing genetic factor in AIMAH by activating the cAMP signaling pathway (81). Furthermore, a high frequency of PDE11A variants has been identified in patients with Carney complex (CNC), especially in those with PRKAR1A mutations. This observation suggests that PDE11A may act as a genetic modifier in the pathogenesis of adrenal and testicular tumors (82). Additionally, inactivating *PDE11A* variants have been associated with familial and bilateral testicular germ cell tumors, underscoring its relevance as a genetic risk factor (**Table 1**) (149, 153).

## 3 Structure and function of PDE10A

### 3.1 Structure

The human PDE10A gene (HSPDE10A1) is located on chromosome 6q26 and encodes a dual-substrate phosphodiesterase that hydrolyzes both cAMP and cGMP (2, 154). PDE10A was first identified in 1999 via bioinformatics approaches. Its expression is predominantly restricted to the central nervous system (CNS), particularly in the medium spiny neurons (MSNs) of the dorsal and ventral striatum, which are integral parts of the basal ganglia (20, 155, 156). Within these neurons, PDE10A is localized in both the cell bodies and dendritic arbors. Low levels of expression have also been reported in other brain regions, including the cortex, hippocampus, and cerebellum (14, 20, 155).

The highest PDE10A expression has been found in the striatum, that have a key role in regulating cAMP/cGMP signaling downstream of dopamine receptor signaling and is critically involved in the changes to gene expression caused by drugs (157). For example, inhibition of PDE10A could be significant in striatal activation and behavioral suppression, representing PDE10A inhibitors useful as antipsychotic agents. The presence of PDE10A in post-synaptic membranes of the medium spiny neurons also enables it to regulate intracellular signaling from glutamatergic and dopaminergic inputs (158). In addition, Birjandi et al. showed that the high expression of PDE10A in the basal ganglia/striatum but not in the prefrontal cortex could be helpful to recover from the striatum using a PDE10A inhibitor (TAK-063), but not cortical stroke, consistent with its brain localization (159). PDE10A inhibitors also could mimic D2 antagonist effects by preferentially activating indirect pathway MSNs, offering potential therapeutic benefits for psychosis, schizophrenia, Tourette syndrome, and other movement disorders (160, 161). Moreover, it has been found that PDE10A integrates into a large protein complex at synaptic membranes, associating with AKAP150, PKA, and NMDA receptors upon PDE10A phosphorylation (162). Low levels of PDE10A expression have been observed in the hippocampus (20); however, Giralt et al. showed that inhibiting the enzyme phosphodiesterase 10A (PDE10A) with the drug papaverine improved spatial and recognition memories in mouse models of Huntington’s disease, possibly via increasing cAMP levels and activating the hippocampal PKA signaling (163). This minimal expression of PDE10A in the hippocampus could, however, counteract its potential benefits in other neurological disorders that should be considered. The PDE10A inhibitor PF-2545920 has been shown to have a pro-epileptic effect by enhancing hippocampal excitability and seizure activity by promoting the trafficking of glutamate receptors (GluA1 and NR2A) to the post-synaptic density and increasing the phosphorylation of GluA1, which strengthens excitatory synapses, leading to synchronized synaptic transmission that can cause seizures (164).

Structurally, like all members of the PDE family, PDE10A contains a catalytic domain at the C-terminus, which is linked to regulatory domains located at the N-terminus. The N-terminal region is essential for dimerization, which is necessary for enzymatic activity, and also provides a platform for alternative splicing (165). At least 18 splice variants of PDE10A have been identified, primarily differing in their N- and C-terminal regions. In humans, PDE10A1 and PDE10A2 are the two primary isoforms, while rodents predominantly express PDE10A2 and PDE10A3. Of these, PDE10A2 is the major isoform expressed in the brain of both species (166).

A unique feature of PDE10A is the presence of GAF domains, which act as regulatory modules capable of binding small molecules, such as cyclic nucleotides (141, 167). In PDE10A, the GAF-B domain adopts a structure consisting of six anti-parallel β-sheets flanked by α-helices, forming a characteristic fold (168). This domain binds cAMP, which allosterically activates the enzyme, making PDE10A the only known mammalian PDE with this function (141).

The amino acid sequence of PDE10A1 and PDE10A2 is identical except for the N-terminus. PDE10A2 contains 789 amino acids, whereas PDE10A1 is 10 residues shorter. This difference results in distinct subcellular localization, with PDE10A2 being associated with the membrane and PDE10A1 residing in the cytosol (166). The catalytic domains of human, rat, and mouse PDE10A are 98% identical in sequence, and the overall amino acid identity is 95% (2, 166).

The crystal structure of the PDE10A2 catalytic domain, comprising 340 amino acids, was published in 2007. The structure contains 15 α-helices, forming a compact topology consistent with other PDEs (169). The active site comprises 11 conserved amino acid residues, including Val678, Thr685, Ala689, Ile692, Tyr693, Met713, Gly725, Gln726, Phe729, Tyr730, and Trp762 (166, 170). This active site is organized into four sub-pockets (171), each playing a critical role in substrate recognition and catalysis. The metal-binding pocket (M) coordinates Zn²^+^ and Mg²^+^ ions, which are essential for catalysis; Zn²^+^ forms strong interactions with histidine and aspartate residues, while Mg²^+^ forms weaker bonds, together activating a water molecule that mediates nucleophilic attack on the phosphodiester bond of the substrate (165, 170). The core pocket (Q) contains an invariant glutamine residue that forms hydrogen bonds with the purine ring of the substrate, providing specificity and stabilizing its orientation for efficient hydrolysis. Adjacent to this, the hydrophobic clamp (H) creates a rigid, hydrophobic environment that accommodates the planar ring structures of substrates or inhibitors, with aromatic residues such as Phe and Ile contributing to binding affinity and selectivity through hydrophobic interactions. Finally, the lid region (L) modulates substrate accessibility and binding by forming a structural lid over the active site, influencing the entry, orientation, and positioning of ligands. Collectively, these sub-pockets act cooperatively, ensuring high substrate specificity and catalytic efficiency while also providing distinct structural features that can be exploited in the design of selective PDE10A inhibitors (166).

Notably, PDE10A also features a unique lipophilic binding pocket not typically found in other PDEs. Within this region, Tyr693 plays a critical role by anchoring ligands via hydrogen bonding, further establishing PDE10A as a druggable target (172). The hydroxyl group of Tyr-693 forms a hydrogen bond with ligands, such as triarylimidazole and pyrazole derivatives, stabilizing their orientation within the pocket (173). This interaction is further reinforced by the structural positioning of the conserved Gln-726, which forms an additional hydrogen bond network with Tyr-693, anchoring the inhibitor effectively. Importantly, this lipophilic pocket is unique to PDE10A due to the presence of Gly-725, which allows access to the pocket, and the deeper M-loop structure, which is longer in PDE10A than in most other PDE isoforms (172). The presence of Tyr-693 in this context provides a structural handle that is not available in other PDEs, except PDE2, where access to the pocket is sterically blocked by a leucine residue (170). Consequently, designing inhibitors that form hydrogen bonds with Tyr-693 while occupying this lipophilic pocket allows for highly selective targeting of PDE10A over other PDE family members. This strategy has been successfully applied in the development of pyrazole and triarylimidazole compounds, which show nanomolar to subnanomolar IC_50_ values and >100–1000-fold selectivity against other PDEs (172).

### 3.2 Function and cellular signaling

The PDE10A is a dual-substrate enzyme that hydrolyzes both cAMP and cGMP, with approximately 20-fold greater affinity for cAMP, making it an ideal target for disorders involving the fronto-striatal circuits (17, 155, 174). This higher affinity enhances phosphorylation of downstream targets such as DARPP-32 and CREB, modulating neuronal excitability, synaptic transmission, and dopaminergic signaling in direct and indirect pathway MSNs, influencing pathway-specific signaling, behavioral outcomes, and therapeutic applications. This makes PDE10A a critical regulator of cAMP-dependent signaling pathways, making PDE10A targeting under the control of cAMP/PKA activity, particularly those involving PKA, which influences other signaling proteins and downstream targets like dopamine- and cAMP-regulated phosphoprotein (DARPP-32) (162, 175). In addition, the mechanism of action of current antipsychotics is reported to be related to activation of the indirect pathway of MSNs in the striatum by the blockade of dopamine D2 receptors, concomitant upregulation of cAMP level, making it essential for monitoring antipsychotic efficacy. PDE10A inhibitors that also cause overactivation of the PDE10A direct pathway could cancel its pharmacological effect (176). In addition, the level of occupancy by PDE10A inhibitors in the striatum has been reported to be directly correlated to an increase in activated cAMP response elements (pCREB), as a marker of neuronal activation (177). Thus, secondary messengers, cAMP and cGMP play critical roles in intracellular signaling, particularly in neuronal pathways involving dopamine and glutamate (178, 179). PDE10A regulates the intracellular concentrations of these nucleotides by catalyzing their hydrolysis to inactive 5’-nucleotides, thereby terminating their downstream signaling cascades (179, 180).

As discussed in the previous section, PDE10A is predominantly expressed in MSNs of the striatum, which are integral to both direct and indirect pathways of the basal ganglia (181, 182). In these neurons, PDE10A critically modulates cAMP/PKA/CREB signaling, which is essential for synaptic plasticity and cognitive function (158, 183). The inhibition of PDE10A results in elevated intracellular cAMP levels, leading to the activation of PKA through its regulatory subunits (184). PKA, in turn, phosphorylates several downstream substrates, including CREB and dopamine- and cAMP-regulated neuronal phosphoprotein 32 kDa (DARPP-32) (185, 186). Phosphorylation of DARPP-32 at threonine-34 (Thr34) converts it into a potent inhibitor of protein phosphatase-1 (PP1), whereas phosphorylation at Thr75 by cyclin-dependent kinase 5 (Cdk5) attenuates PKA signaling (187, 188). In line with these mechanisms, further insights from genetic and pharmacological studies have clarified how specific phosphorylation states of DARPP-32, particularly at Thr34 and Thr75, dictate the differential responses of MSNs to PDE10A inhibition. The phosphorylation of DARPP-32 at Thr75 by Cdk5 converts it into an endogenous PKA inhibitor, dampening cAMP-mediated signaling (183, 188). However, studies in Thr75Ala mutant mice, as well as experiments using Cdk5 inhibition with roscovitine, revealed no significant changes in the D1/D2 imbalance of PDE10A inhibition responses (189). These findings indicate that Thr75 phosphorylation does not critically determine the differential MSN responses to PDE10A inhibition. Taken together, these results highlight that PDE10A inhibition primarily alters neuronal signaling homeostasis through Thr34 phosphorylation of DARPP-32, leading to enhanced PKA signaling and PP1 inhibition in D2 MSNs. This mechanism explains the selective responsiveness of indirect pathway MSNs and supports the role of DARPP-32 as a key molecular switch integrating PDE10A-dependent cAMP/PKA signals in the striatum (175, 190–193). The experimental evidence, including studies using the PDE10A inhibitor papaverine, indicates that these functional outcomes are predominantly mediated by cAMP/PKA signaling, with minimal effects on cGMP/PKG pathways (17, 183). Thus, the higher affinity of PDE10A for cAMP shifts the intracellular signaling balance in MSNs toward cAMP-dependent pathways, providing a mechanistic basis for its effects on striatal function. Through these mechanisms, PDE10A inhibition alters the phosphorylation state of ion channels such as AMPA and GABA-A receptors, thus influencing neuronal excitability and synaptic transmission (194, 195).

As highlighted in recent studies, phosphorylation of DARPP-32 at Thr34 by PKA enhances its ability to inhibit PP1, thereby sustaining PKA-mediated phosphorylation of multiple downstream substrates, including ion channels and receptor subunits (196). This modulation has direct functional implications for receptor sensitivity and neuronal excitability. For example, disruption of DARPP-32 significantly attenuates the phosphorylation of GABAA_AA​ receptor β1/β3 subunits following D1 receptor stimulation, leading to reduced efficacy of D1 receptor coupling to GABAA_AA​ receptor currents (197). Functional assays in DARPP-32 knockout mice demonstrated that at low levels of D1 receptor stimulation, the absence of phospho-DARPP-32 markedly diminishes the ability of D1 agonists (e.g., SKF 81297) to modulate GABA-evoked currents. In contrast, at higher agonist concentrations, this deficit can be partially overcome (197). These findings indicate that phospho-DARPP-32 inhibition of PP1 is particularly critical under conditions of submaximal dopaminergic input, where it amplifies the signal-to-noise ratio of D1 receptor signaling to GABAA_AA​ receptors.

Beyond GABA_A_​ receptors, the phosphorylation state of DARPP-32 may also influence other PKA-regulated ion channels such as AMPA receptors, L-type Ca^2+^ channels, and NMDA receptors, thereby modulating synaptic plasticity and excitability (194, 198, 199). These effects underscore the role of DARPP-32 as a central integrator of dopaminergic and glutamatergic signaling, with PDE10A inhibition indirectly affecting ion channel phosphorylation states through the DARPP-32/PP1 regulatory axis (200).

Given its regulatory role in critical signaling pathways, PDE10A is implicated in the pathophysiology of multiple neuropsychiatric and neurodegenerative disorders, including schizophrenia, HD, PD, and Alzheimer’s disease (AD) (201, 202). In schizophrenia, PDE10A is implicated due to its high expression in dopaminoceptive MSNs and its role in modulating both dopaminergic and glutamatergic signaling. PDE10A inhibition increases cAMP levels, activating D1-like signaling in the direct pathway and mimicking D2 receptor antagonism in the indirect pathway (185, 190–193). These effects support its therapeutic potential in mitigating both positive and negative symptoms of schizophrenia (185, 193, 201). In PD, PDE10A expression is downregulated in dopaminergic-depleted regions such as the striatum, correlating with motor dysfunction severity (4, 201, 203, 204). Interestingly, PDE10A levels are upregulated in the nucleus accumbens following dopamine loss, potentially contributing to disease progression [53]. In AD, downregulation of the adenylyl cyclase/cAMP/PKA pathway and inhibition of CREB by β-amyloid plaques contribute to cognitive deficits (201, 205). In HD, a neurodegenerative disorder marked by loss of striatal GABAergic MSNs, PDE10A expression is significantly reduced in advanced stages (142). Despite this, acute inhibition of PDE10A in HD mouse models enhanced corticostriatal input and increased phosphorylation of CREB, suggesting a compensatory therapeutic mechanism (206, 207).

PDE10A is not only involved in CNS disorders but also plays a role in peripheral cancers. It is highly expressed in NSCLC and CRC compared to normal tissues (5, 6). Therefore, inhibiting PDE10A activates the cGMP/PKG pathway, downregulates β-catenin, and promotes apoptosis through caspase and PARP activation (5, 6, 11, 208). It also suppresses MEK/ERK and RAF/MAPK signaling, highlighting its tumor-suppressive potential (5, 11). In the subsequent sections, we will examine the effect of PDE10A in various malignancies.

## 4 PDE10A as a cancer biomarker

PDE10A has emerged as a context-dependent modulator of tumorigenesis. PDE10A appears to act as either a tumor suppressor or an oncogene based on tissue type and the surrounding tumor microenvironment. This dual role highlights its potential as a valuable biomarker for diagnosis, prognosis, and targeted therapy in various cancers (6).

In GBM, PDE10A has been identified as a haploinsufficient tumor suppressor located at chromosomal locus 6q27. Combined CRISPR/Cas9 data, human spatial transcriptomic data, and human and mouse RNA sequencing data led to the nomination of PDE10A as a potential haploinsufficient tumor suppressor in the 6q27 region. Loss of *PDE10A* in GBM correlates with poor prognosis and aggressive tumor phenotypes across multiple GBM cohorts. Mechanistically, the RCAS/tv-a mouse model demonstrates that PDE10A depletion enhances tumorigenicity by activating the PI3K/AKT pathway independent of PTEN. It has been shown that PDE10A suppression leads to increased phosphorylation of AKT (pAKT) and PI3K (p-PI3K) without altering total AKT and PI3K levels, indicating activation of the PI3K/AKT pathway. However, PTEN levels were not different between the PDE10A knockdowns and control, showing that PDE10A suppression activates the PI3K/AKT pathway independent of PTEN (209). This is accompanied by a phenotypic shift from proneural to mesenchymal transcriptional state, a hallmark of treatment-resistant GBM (209, 210).

In contrast to GBM, epithelial ovarian cancer, CRC, and NSCLC, showed to have a higher PDE10A expression that contributes to tumor progression, therapy resistance, and reduced patient survival (6, 12, 211). In ovarian cancer, while PDE10A mRNA may be downregulated, elevated protein levels in tumor tissues and cell lines (such as OVCAR8, SKOV3) indicate post-transcriptional regulation (12). High PDE10A expression was also reported to be related to a significant poor overall survival (12).

In NSCLC, elevated PDE10A expression has been observed at both mRNA and protein levels, particularly in lung adenocarcinoma. Its predominant cytoplasmic and membranous localization reflects a tumor-specific pattern (212). Pharmacologic inhibition using selective inhibitors such as Pf-2545920 activates the cGMP/PKG axis, suppresses β-catenin, and inhibits MAPK signaling, leading to decreased proliferation and increased apoptosis (5, 212).

Consistent with these findings, the antitumor activity of PDE10A inhibition in colorectal cancer appears to be primarily mediated through activation of the cGMP/PKG signaling axis rather than the cAMP/PKA pathway. Mechanistically, PKG exerts a dual repressive influence on oncogenic β-catenin signaling. First, it suppresses transcription of the CTNNB1 gene, thereby reducing both β-catenin mRNA expression and protein abundance. Second, PKG promotes the nuclear retention of FOXO4, which sequesters β-catenin and consequently diminishes its availability for TCF-dependent transcriptional activation. Notably, these effects occur independently of proteasome-mediated β-catenin degradation via canonical phosphorylation at Ser33/37/Thr41 (213). By contrast, PKA signaling has been shown to stabilize β-catenin through phosphorylation at Ser552 and Ser675, which enhances its nuclear localization and transcriptional activity (214). These observations indicate that the growth-suppressive effects of PDE10A inhibition in colorectal cancer cells are critically dependent on PKG-mediated downregulation of β-catenin signaling, a mechanism not effectively reproduced by PKA activation.

The PDE10A rs12660420 variant is associated with a higher risk of developing tobacco-related NSCLC. Interestingly, elevated PDE10A expression is linked to better clinical outcomes in patients with early-stage NSCLC (211). In CRC, PDE10A is variably regulated across the neoplastic continuum from precancerous lesions to advanced metastatic stages (6, 215). *In vitro*, inhibition of PDE10A reduces CRC cell viability, induces G2/M cell cycle arrest, activates caspase-3, and promotes apoptosis (6, 11). Notably, PDE10A is also overexpressed in histologically normal mucosa of CRC patients, suggesting potential as an early detection biomarker (6, 156). Furthermore, decreased PDE10A expression is associated with an EMT phenotype and metastatic disease in colon adenocarcinoma (215). In prostate cancer (PCa), somatic mutations in PDE10A are restricted to tumor tissues and have lower expression in regular counterparts. Upregulation of PDE10A disrupts the cAMP/cGMP balance, elevating pCREB/CREB ratios and activating CREB-dependent oncogenic transcription programs (216). In metastatic gastric cancer, deleterious mutations in PDE10A have been identified exclusively in peritoneal metastases, implicating this gene in metastatic transition and phenotypic plasticity (217).

Beyond solid tumors, paraneoplastic neurological syndromes (PNS) have been associated with anti-PDE10A IgG antibodies. These have been detected in patients presenting with movement disorders and concurrent malignancies such as lung and renal cancers. PDE10A may function as an onconeural antigen, potentially triggering T-cell responses, especially in patients undergoing immune checkpoint blockade (218). Additionally, *PDE10A::BRAF* fusions have been reported in rare pediatric sarcomas. These fusion drives the aberrant activation of the MAPK pathway, promoting tumorigenesis. Such alterations underscore the therapeutic relevance of PDE10A in precision oncology for pediatric mesenchymal tumors (219, 220).

In summary, PDE10A demonstrates context-specific roles across various cancer types. The differential expression of PDE10A and its regulatory influence on oncogenic pathways, such as the PI3K/AKT, MAPK, and β-catenin pathways, underscores its role in cancer progression. In addition, overexpression of PDE10A in cancer cells and tumors compared to normal cells and normal tissues has been observed (221). Loss of the PDE10A gene locus (6q27) could be considered an independent poor prognostic indicator in IDH wild-type glioblastoma, showing lower overall survival (209). Similarly, PDE10A overexpression in ovarian cancer has been reported to be in association with worse overall survival and upregulation of oncogenic pathways (Wnt/β-catenin, RAS/MAPK) (12). In addition, a study by Zekeridou et al. introduces a novel autoantibody, PDE10A IgG, as a biomarker for a rare paraneoplastic neurological syndrome that affects older adults with cancer (particularly lung, renal, and pancreatic carcinomas) and is characterized by hyperkinetic movement disorders (218). These associations with prognosis and treatment response support its potential as a biomarker for diagnosis, prognosis, and targeted therapy in oncology for different tumors (**Table 2**).

**Table 2:** An overview of PDE10A targeted or used as a biomarker in each cancer.

Cancer	Author	Design	Outcome	Reference
Ovarian	Borneman	*In vivo* and *in vitro*	-Pf-2545920 and MCI-030 inhibitors suppress ovarian cancer cell growth by inducing cell cycle arrest and apoptosis through the dual inhibition of Wnt/β-catenin and EGF-mediated RAS/MAPK/AKT signaling pathways.	(12)
	Chen	*In vivo* and *in vitro*	-PDE10A contributed to DOX-induced mouse cardiomyocyte death via increasing Top2β expression, mitochondrial dysfunction, and DNA damage by antagonizing cGMP/PKG signaling. -PDE10A contributed to mouse cardiomyocyte atrophy via potentiating FoxO3 signaling via both cAMP/PKA and cGMP/PKG dependent signaling. -PDE10A inhibition alleviated DOX-induced myocardial atrophy and apoptosis. -PDE10A inhibition increased the death, potentiated the effect of DOX on various human cancer cells.	(222)
Colorectal cancer	Li	*In vivo* and *in vitro*	-PDE10 inhibition selectively activates cGMP/cGMP-dependent protein kinase signaling to suppress β-catenin levels and T-cell factor (TCF) transcriptional activity in colon tumor cells.	(6)
	Lee	*In vivo* and *in vitro*	PDE10 knockdown reduces the growth inhibitory activity of Pf-2545920. -Pf 2545920 inhibits the translocation of β-catenin to the nucleus. -Pf-2545920 increases cGMP and cAMP levels, and activates PKG and PKA, reduces β-catenin-mediated transcription of survival proteins	(11)
	Li	*In vitro*	-Combined inhibition of suppresses β-catenin, TCF transcriptional activity, and the levels of downstream targets, cyclin D1 and surviving.	(223)
	Lee	*In vivo* and *in vitro*	- ADT 061 activated cGMP/PKG signaling, induced phosphorylation of oncogenic β-catenin, inhibited Wnt-induced nuclear translocation of β-catenin, and suppressed TCF/LEF transcription, suppressed the formation of colon adenomas	(10)
	Arowa	*Ex vivo*	-Loss of PDE10A associated with EMT transcriptional state and metastatic disease.	(215)
Glioblastoma	Kopanitsa	*In vitro*	-PF 2545920, PQ10, and papaverine decreased GBM cell proliferation.	(224)
	Nuechterlein	*In vivo* and *in vitro*	-decreased PDE 10A expression led to increased PI3K/AKT signaling and proneural-to-mesenchymal transition	(209)
Non-small cell lung cancer	Fusco	Clinical trial	- correlated with survival in patients with stage I–II NSCLC	(211)
	Shen	*In silico*	-p.Pro360Ala on PDE10A played a potential oncogenic role in mediating tumorigenesis	(212)
Lung	Zhu	*In vitro*	-PQ10 induces apoptosis by increasing intracellular cGMP levels and activating PKG to suppress oncogenic β-catenin and MAPK signaling.	(5)
Gastric	Liu		-The 9 genes including ERBB4, ZNF721, NT5E, PDE10A, CA1, NUMB, NBN, ZFYVE16, and NCAM1 were only mutated in metastasis.	(217)
Spindle Cell Sarcoma	Hughes	Case report	-An ETV6-NTRK3 fusion-negative spindle Cell sarcoma with IFS-like morphology, subjected to genomic profiling, revealed a PDE10A-BRAF fusion.	(219)
Paraneoplastic neurologic autoimmunity	Zekeridou	Clinical trial	-PDE10A may function as an onconeural antigen, potentially triggering T-cell responses, especially in patients undergoing immune checkpoint blockade	(218)

PDE10A, Phosphodiesterase 10A; IFS, Infantile/congenital fibrosarcoma; NSCLC, Non-small cell lung cancer; PI3K, phosphoinositide 3-kinase; TCF, T-cell factor; DOX, Doxorubicin.

## 5 PDE10A inhibition in cancer

Elevated PDE10A expression has been documented in multiple cancer types, including colorectal cancer (CRC) and non-small cell lung cancer (NSCLC) (5, 6). Notably, selective PDE10AIs significantly attenuate cell proliferation in several CRC cell lines (HT29, SW480, and HCT116). This antiproliferative effect is reported to be mediated via the cGMP/PKG signaling axis, leading to phosphorylation and functional suppression of β-catenin. Consequently, PDE10AIs offer a novel molecular strategy to inhibit β-catenin-driven transcriptional activity in tumor cells (225). Therefore, Pharmacological inhibition of PDE10A has emerged as a promising strategy in cancer therapeutics due to its selective overexpression in malignant tissues and minimal expression in corresponding normal cells (6–12,). Multiple small-molecule inhibitors have been developed to target PDE10A, including papaverine, PQ-10, PF-2545920 (MP-10), TP-10, ADT-061, and MCI-030. These agents have shown potent anti-tumor activity in preclinical models of such as ovarian, colorectal, lung, and breast cancers. These inhibitors function by elevating intracellular levels of cyclic nucleotides (cAMP and cGMP), leading to activation of the downstream effectors PKA and PKG. The consequent modulation of signaling cascades results in the suppression of key oncogenic pathways, such as the Wnt/β-catenin, RAS/MAPK, and PI3K/AKT pathways (7, 9, 10, 12, 226). In this context, a study conducted by Kopanitsa et al. examines the effects of 28 PDE inhibitors, including PF-2545920, PQ10, and papaverine, as PDE10A inhibitors on human U87MG, A172, and T98G GBM cells. The results demonstrated that pharmacological inhibition of PDE10A using PF-2545920, particularly in combination with the PDE5 inhibitor MY-5445 and multidrug resistance-associated protein 1 inhibitor reversan, leads to synergistic suppression of GBM cell proliferation *in vitro*, suggesting a promising low-toxicity therapeutic strategy (224).

Moreover, PF-2545920 and MCI-030 significantly inhibited cell proliferation and induced apoptosis in ovarian cancer cell lines (SKOV3, OV-90, and OVCAR3). These effects were associated with increased phosphorylation of VASP at Ser157 and Ser239, indicating activation of PKA and PKG pathways. These treatments also led to decreased nuclear localization of β-catenin and reduced expression of β-catenin target genes, including c-MYC, survivin, and cyclin D1 (**Table 2**) (7, 226). MCI-030, a sulindac derivative, demonstrated high potency with an IC_50_ of ~0.5 µM and achieved stable plasma and ovarian tissue concentrations (~2 µM) after oral administration in murine models, with an excellent safety profile (7). Similarly, ADT-061, another sulindac-derived non-COX-inhibitory compound, selectively targeted colon tumor cells (IC_50_ = 0.3–0.5 µM), sparing normal colonocytes. It enhanced intracellular cGMP levels, activated PKG, and inhibited Wnt/β-catenin signaling by reducing nuclear β-catenin translocation (9). ADT-061 demonstrated chemopreventive efficacy in *Apc^^+/Min-FCCC^* mice by significantly reducing colorectal adenoma burden without observable toxicity (**Table 2**) (8, 10).

**Figure 1:**
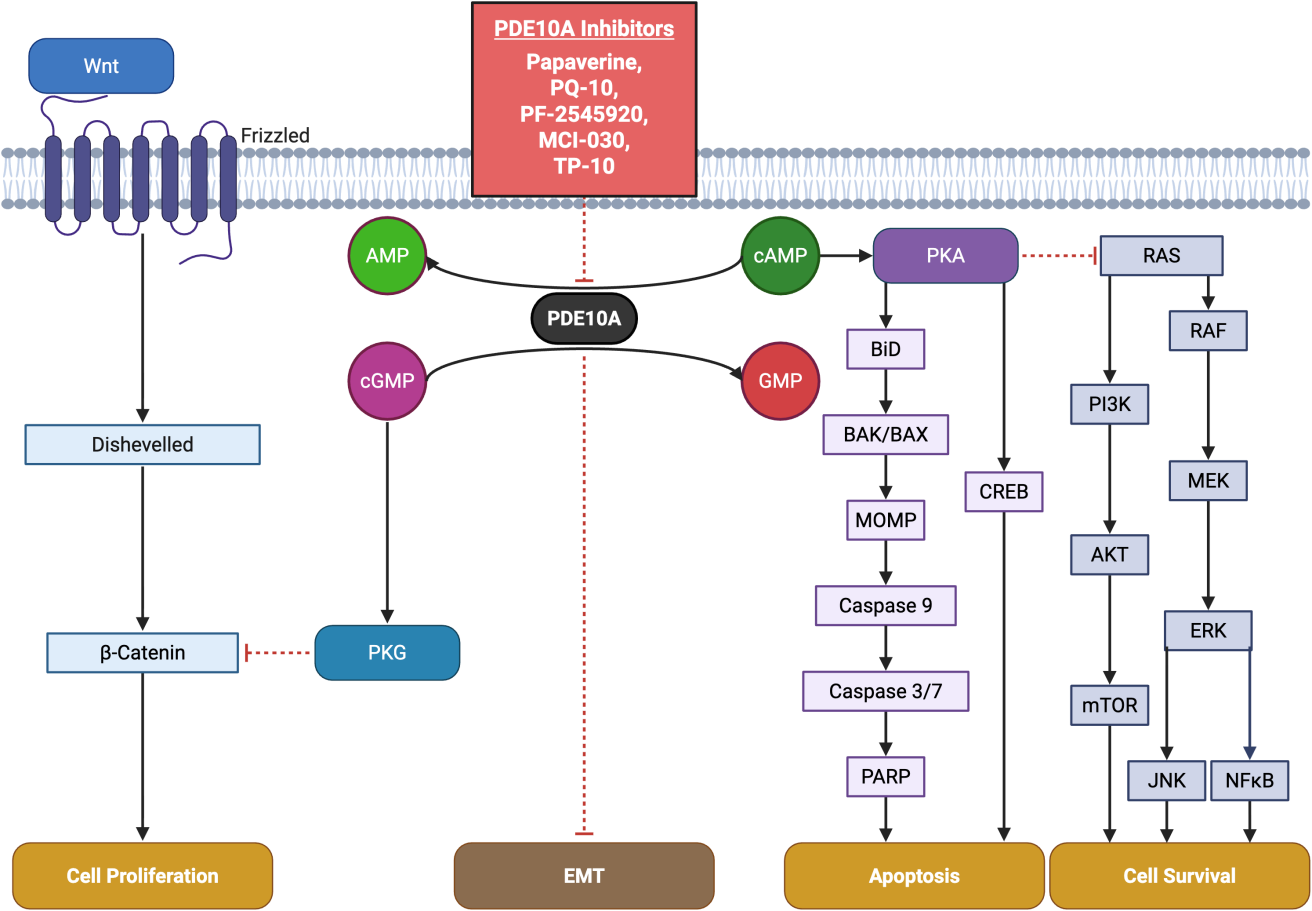
Key pathways involving PDE10A in cancer and pharmacological targeting by small-molecule inhibitors. The schematic highlights critical PDE10A-mediated signaling nodes including cAMP/cGMP, PKA, PKG, and cross-talk with CREB, MAPK, PI3K or Wnt/β-catenin pathways implicated in tumor proliferation, survival, and apoptosis. Known PDE10A inhibitors including TP-10, MCI-030, papaverine, PQ-10, and PF-2545920 and their chemical structures are shown, emphasizing their potential therapeutic for cancer.

In colon cancer models, PDE10 inhibitors comprising PF-2545920, papaverine, and PQ-10, induced G1-phase cell cycle arrest, inhibited proliferation, and promoted apoptosis, with reduced cytotoxic effects on normal epithelial cells. Similar findings were reported in colon cancer cell lines (HCT116, HT29), where siRNA-mediated PDE10A knockdown mimicked pharmacological inhibition, resulting in decreased cell viability, caspase-dependent apoptosis, and reduced DNA synthesis (6). Notably, both pharmacological inhibition and siRNA approaches selectively activated the cGMP/PKG signaling pathway in cancer cells. This was evidenced by increased phosphorylation of VASP at Ser239, with minimal effect on cAMP/PKA signaling. This functional specificity likely reflects a tumor-context preference for cGMP regulation mediated by PDE10A. It may be attributed to the kinetic properties of the enzyme or the compensatory degradation of cAMP by other PDE isoforms in normal tissues (10). Moreover, stable knockdown of PDE10A using lentiviral shRNA vectors impaired the anchorage-independent growth and colony formation of colon tumor cell lines such as HT-29, SW-480, and HCT-116, reinforcing the critical role of PDE10A in sustaining malignancy (11). At the molecular level, PDE10A inhibition resulted in transcriptional repression of β-catenin target genes, including cyclin D1 and survivin. This effect was likely mediated by nuclear PKG interfering with TCF/LEF transcriptional activity and downregulating CTNNB1 gene expression (**Table 2**) (10). Collectively, these findings highlight the therapeutic potential of PDE10A inhibition in specific cancer types. The development and preclinical evaluation of selective inhibitors, such as ADT-061 and MCI-030, underscore their efficacy in suppressing tumor growth and promoting apoptosis, while exhibiting minimal toxicity (**Figure 1**).

However, this finding appears to contrast with the results of Nuechterlein et al, who introduce PDE10A as a potential haploinsufficient tumor suppressor in the 6q27 region. Moreover, they developed a PDE10A-suppressed GBM model using the RCAS/tv-a system in mice, which caused an aggressive glioma phenotype, promoted a proneural-to-mesenchymal transition, and increased resistance to chemo- and radiotherapy. Cell culture analysis also showed that reduced expression of PDE10A activates the PI3K/AKT pathway, contributing to glioma progression and temozolomide resistance (209). This discrepancy may be attributed to differences in experimental models (pharmacologic inhibition vs. genetic knockout), the degree and duration of PDE10A suppression. This suggest that in contrast to acute drug inhibition using small molecules and inhibitors that decrease proliferation of GBM cells, the long-term knockdown may allow for compensatory PI3K/AKT signaling that promotes tumor growth, make it vulnerable to PI3K inhibition (209). Critically, the anti-tumor efficacy of PDE10A inhibitors like PF-2545920 and MCI-030 occurs at concentrations that significantly elevate cyclic nucleotides without immediately activating PI3K/AKT feedback loops in responsive tumors. In addition, combination strategies with PI3K/AKT pathway inhibitors may help mitigate compensatory activation, as seen in GBM models where PI3K inhibitors blocked AKT activation induced by PDE10A suppression (209). Accordingly, it also increases the importance of existence and establishing a therapeutic window, describing the dose and period of time of treatment, which may be based on tumor PDE10A expression and PI3K/PTEN status, to maximize efficacy while minimizing the risks of compensatory signaling.

Moreover, pharmacological inhibitors, such as PF-2545920, ADT-061, and MCI-030, selectively elevate intracellular cAMP and/or cGMP, activating PKA or PKG pathways, in ovarian, colorectal, and other cancer models suggesting a tumor-specific preference for cyclic nucleotide regulation, possibly due to kinetic properties of the enzyme or compensatory cAMP degradation by other PDE isoforms. This could be related to the context-dependent manner of PDE10A inhibition, which modulates cAMP and cGMP levels accordingly, leading to differential activation of PKA and PKG across various cancer models. Moreover, this contrast finding may be due to tissue-specific expression of PDE10A isoforms (higher level of cAMP in ovary) (182, 227), baseline cyclic nucleotide levels, and cross-talk with other pathways.

**Figure 2:**
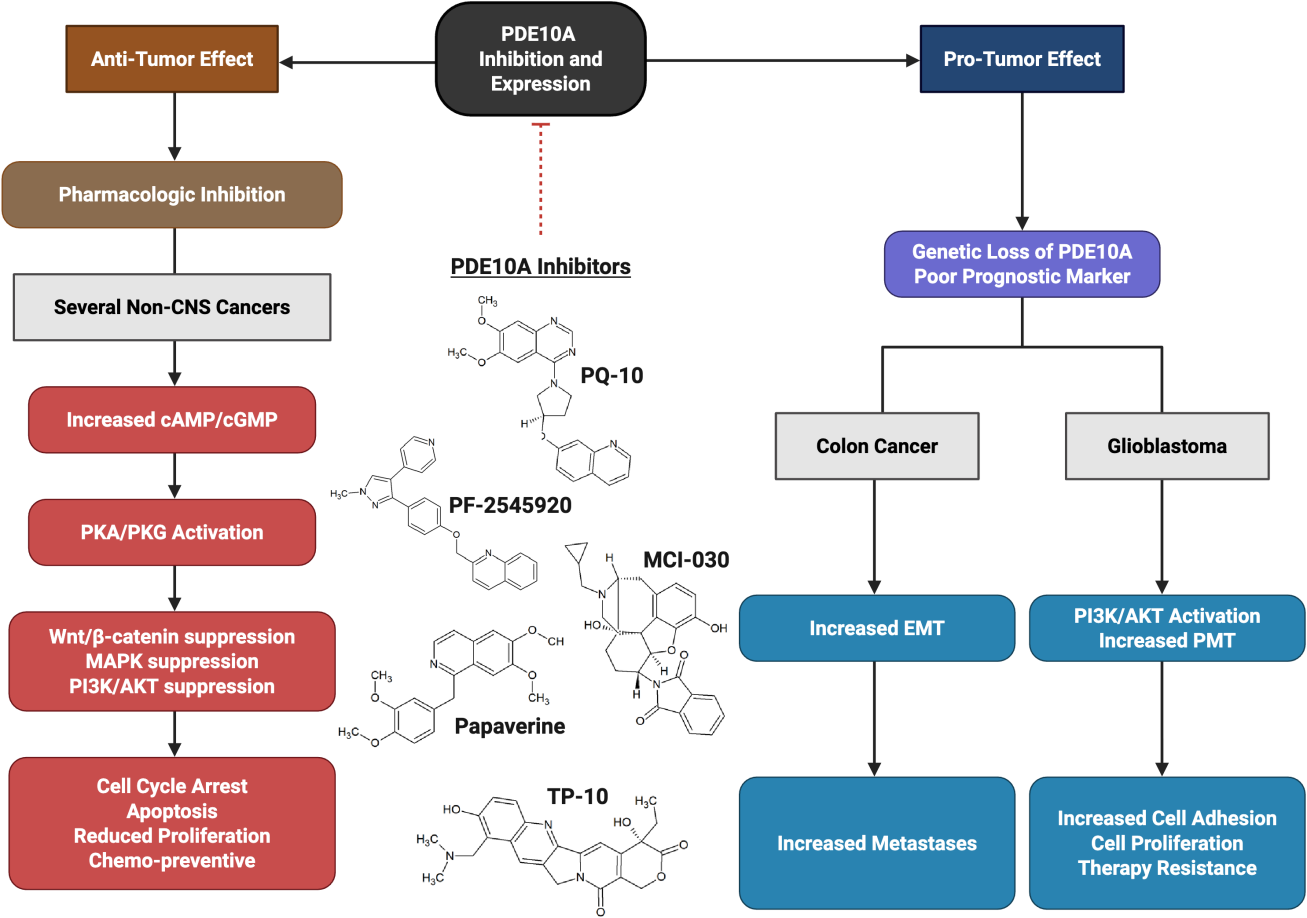
The dual role activity of PDE10A. Tumor promoting role in glioblastoma (GBM) via activation of PI3K/AKT pathway and enhancing proneural–mesenchymal transition (PMT). Anti-tumor activity (most cancers) after inhibition with PDE10A Inhibitors (such as TP-10, MCI-030, papaverine, PQ-10, and PF-2545920) via increase in Cyclic adenosine monophosphate (cAMP) and cyclic guanosine monophosphate (cGMP), and suppressing Wnt/β-catenin, RAS/MAPK, and PI3K/AKT.

Therefore, while PDE10A inhibition shows promise as a therapeutic strategy under controlled pharmacologic conditions, these divergent outcomes emphasize the need for tumor-specific molecular profiling and careful consideration of potential pro-tumorigenic consequences when translating PDE10A-targeted therapies to the clinic (**Figure 2**).

## 6 Future direction

Preclinical studies have consistently demonstrated the antitumor efficacy of PDE10A inhibitors across various cancer models. However, clinical translation remains limited. Several PDE10A inhibitors have entered Phase I and II clinical trials for non-oncologic indications, such as schizophrenia (ClinicalTrials.gov Identifiers: NCT00570063, NCT02477020, NCT02019329, NCT01568203), and have shown favorable safety profiles (228). Despite this, robust data on pharmacokinetics, optimal dosing strategies, and long-term effects in clinical trials of oncology are lacking. Further clinical studies are necessary to determine the therapeutic viability of these approaches in cancer settings. In addition, PDE10A exhibits context-dependent roles within tumor biology. In GBM, it may function as a tumor suppressor (209), whereas in CRC, NSCLC, and ovarian cancers, it contributes to tumor progression (6, 12, 211). The same dual role has been observed and reported in this review article in other PDEs including PDE1, PDE2, PDE4, and PDE7 that could complicate therapeutic development and underscores the need for molecular studies to elucidate tumor-specific signaling contexts and microenvironmental factors that modulate PDE10A activity. Precision targeting requires a clear understanding of these context-dependent mechanisms.

Resistance to PDE10A-targeted monotherapy also presents another significant challenge. Adaptive signaling through compensatory pathways may limit long-term efficacy. Rational combination therapies that co-target synergistic pathways, such as the MAPK pathway, could enhance treatment outcomes (5, 212). Multi-omics integration and computational modeling provide strategic tools for predicting effective drug combinations and resistance mechanisms. Non-invasive imaging and molecular diagnostics should be developed to guide clinical decision-making and improve precision medicine approaches.

Beyond oncology, PDE10A inhibition holds promise in reducing unintended toxicities. Preclinical evidence suggests protective effects against doxorubicin-induced cardiotoxicity, indicating potential value in cardio-oncology. A broader exploration of PDE10A inhibitors in other disease domains may expand their therapeutic utility (229).

## 7 Conclusion

PDE10A plays a multifaceted role in both physiological and pathological contexts. Its dual function, as an oncogene in certain cancers, such as CRC, and a tumor suppressor in GBM, highlights the complexity of its molecular regulation and context-dependent activity as seen in other PDEs including PDE1, PDE2, PDE4, and PDE7. Structural insights into its GAF and catalytic domains have expanded our understanding of its unique enzymatic profile. The emerging data position PDE10A as a potential biomarker and therapeutic target, especially in CNS disorders and various malignancies, including GBM, NSCLC, and CRC. Selective PDE10A inhibitors demonstrate promise in preclinical studies, although clinical translation remains in its infancy. Further investigation is warranted to elucidate tissue-specific mechanisms and to develop targeted therapies that utilize the biological functions of PDE10A. In addition, it’s suggested to examine the tissue-specific expression of PDE10A, baseline cyclic nucleotide levels, cross-talk with other pathways, differences in the degree and duration of PDE10A suppression, and the interplay between PDE10A-mediated cyclic nucleotide signaling and compensatory oncogenic pathways for an effective therapy. Additionally, studying the protein expression along with mRNA expression in necessary to consider post-transcriptional regulation. Moreover, it seems a lack of study comparing acute and chronic PDE10A inhibition with genetic tools or pharmacological inhibition with both toxic and non-toxic functional dose to make sure these contrast results are not related to dose, method or duration of suppression. In addition, investigating and considering all these factors seems necessary before any intervention on PDEs, bringing to us the importance of personalized medicine for patients suffering from cancer.
